# A genetic study of autism in Costa Rica: multiple variables affecting IQ scores observed in a preliminary sample of autistic cases

**DOI:** 10.1186/1471-244X-5-15

**Published:** 2005-03-21

**Authors:** L Alison McInnes, Patricia Jiménez González, Elina R Manghi, Marcela Esquivel, Silvia Monge, Marietha Fallas Delgado, Eduardo Fournier, Pamela Bondy, Kathryn Castelle

**Affiliations:** 1 Mount Sinai School of Medicine, New York, New York, USA; 2 Hospital Nacional de Ninos "Dr Sáenz Herrera";CCSS, Child Developmental and Behavioral Unit, San José, Costa Rica; 3 University of Illinois at Chicago, Chicago, Illinois, USA

## Abstract

**Background:**

Autism is a heritable developmental disorder of communication and socialization that has not been well studied in Hispanic populations. Therefore, we are collecting and evaluating all possible cases of autism from a population isolate in the Central Valley of Costa Rica (CVCR) for a clinical and genetic study.

**Methods:**

We are assessing all subjects and parents, as appropriate, using the newly translated Spanish versions of the Autism Diagnostic Interview-Revised (ADI-R) and the Autism Diagnostic Observation Schedule (ADOS) as well as tests of intelligence and adaptive behavior. Detailed obstetric and family medical/psychiatric histories are taken. All cases are tested for Fragile X and will be extensively evaluated for cytogenetic abnormalities.

**Results:**

To date we have obtained clinical evaluations on over 76 cases of possible autism referred to our study and report data for the initial 35 complete cases. The mean age of the probands is 6.7 years, and 31 of the 35 cases are male. Twenty-one of the cases have IQs <50 and only 6 cases have IQs ≥ 70. Over half of the mothers had complications during pregnancy and/or delivery. No cases have tested positively for Fragile X or PKU. Chromosomal G-banding is not yet complete for all cases.

**Conclusion:**

Diagnostic data gathered on cases of autism in the CVCR using Spanish versions of the ADI-R and ADOS look similar to that generated by studies of English-speaking cases. However, only 17% of our cases have IQs within the normal range, compared to the figure of 25% seen in most studies. This result reflects an ascertainment bias in that only severe cases of autism come to treatment in the CVCR because there are no government-sponsored support programs or early intervention programs providing an incentive to diagnose autism. The severity of mental retardation seen in most of our cases may also be exaggerated by the lack of early intervention programs and the use of IQ tests without Costa Rican norms. Still, we must formally train healthcare providers and teachers to recognize and refer autistic cases with normal or near normal IQs that are not seen in treatment.

## Background

Autism (MIM 209850) is a heritable neurodevelopmental disorder characterized by delayed language acquisition, impaired socialization, and repetitive or stereotyped behaviors. Frequent associated features include sensory abnormalities and lack of motor coordination. The recurrence risk for siblings is approximately 30 times that of the general population, and twin studies have documented a higher concordance rate in monozygotic (60–91%) than in dizygotic twins (0–6%) [1]. According to a summary of 32 epidemiological studies, the male:female ratio is ~4:1, and up to 75% of autistic subjects are mentally retarded to some degree [2]. Associated medical disorders were estimated to be present in ~6.4 % of autistic individuals in that review. Four studies also indicate that siblings of autistic probands are at increased risk for Pervasive Developmental Disorder Not Otherwise Specified (PDDNOS) and Asperger's Disorder, suggesting that these disorders exist on a continuum [3-6]. In fact, a mutation in the *Neuroligin 3 *gene on Xq13 has been identified in a sibship where one brother has autism and the other has Asperger's [7]. This mutation was inherited from the mother and changes a highly conserved arginine residue to cysteine (R451C), resulting in retention of the protein in the endoplasmic reticulum and reduced binding with its presynaptic partner beta neurexin-1 [8]. Although mutations in this gene may not explain a substantial portion of the population risk for autism, the finding provides definitive proof for the genetic basis of autism spectrum disorders (ASD) and encouragement for ongoing genetic studies such as ours.

Multiple genes and environmental factors likely contribute to the etiology of autism, making it difficult to isolate disease genes. There is a substantial fall-off in risk of autism for second- and third-degree relatives of autistic probands [9], and some predict that alleles of 10 to 100 or more genes, each of small effect, may underlie the autistic phenotype [10,11]. Hirschprung's disease, or congenital megacolon, is the prototype of a complex genetic trait and displays an inheritance pattern that could well be representative of autism. To date, there are more than nine susceptibility genes for this disorder, and different combinations of alleles interact to produce the phenotype. The penetrance of Hirschprung's disease is low, and the phenotype varies widely in severity within and among families. This pattern is also seen in the family mentioned above, where a sibling with autism and a sibling with Asperger's share at least one identical genetic determinant.

Two principle strategies have been used to try to limit the genetic heterogeneity underlying such complex phenotypes. One proven method is to identify more genetically homogeneous subphenotypes. With respect to autism, language delay and obsessive compulsive behaviors are traits that segregate within both affected and unaffected members of a family [12,13]. In practice, subsetting samples according to the presence of language delay has strengthened evidence for linkage to the same regions on chromosomes 2q and 7q in several different studies [14-17].

Another, albeit less successful, means of reducing genetic heterogeneity is to study these disorders in isolated founder populations. These populations are derived from a small number of original founders and then grow exponentially, in isolation, such that the small number of disease alleles introduced by the founders will be widely distributed in the present day. For instance, genes for Hirschprung's disease have been identified in the genetically isolated population of the old order Mennonites, although some degree of heterogeneity was present even in this population [18].

The CVCR is another well known founder population widely studied by geneticists interested in neuropsychiatric disorders and other complex traits like asthma [19]. The CVCR was founded ~15 generations ago by a small number of families from southern Spain, and the local, predominantly female, Amerindians. These assertions, based largely on civil and church registries, have been confirmed at the genetic level using haplotype data from mitochondrial DNA (mtDNA), Y-chromosome, and autosomal markers [20]. Mitochondrial data indicate that the four major Native American mtDNA lineages are enriched in females from the CVCR. Data from the Y chromosome shows that the male ancestry in CVCR is about 0.86 European, with a small contribution from West African and Amerind populations. Importantly, an isolated founder population in Antioquia, Colombia shares the same demographic history as the CVCR, and autism case collection is currently underway in that region using the same study protocol as ours. The only published studies of ASD in an isolated population are ongoing in Finland [21-23]. A comparison of gene diversity between Finland and the CVCR shows that they are similar at the autosomal level [20].

Previously, genetic studies of autism were not undertaken in the CVCR because the Autism Diagnostic Interview-Revised (ADI-R) [24] and the Autism Diagnostic Observation Schedule (ADOS) [25], the standard research instruments used to diagnose autism, were available only in English. Furthermore, application of the ADI-R and ADOS for research studies requires formal certification training in English. The ADI-R is a structured diagnostic interview administered to parents that evaluates social abilities, verbal and non-verbal communication, and repetitive and stereotyped behaviors in possibly autistic children. In our population, the test takes anywhere from 2 to 3.5 hours, depending upon the parent's level of awareness. The ADOS is a semi-structured standardized assessment of social interaction, communication, and play that can distinguish autism and PDDNOS from non-spectrum disorders. The test is 30 minutes long, and one of four modules is administered to the case, according to their level of expressive language. Fortunately, a Spanish-language version of the ADI-R is now available, and our group participated in the recent translation of the ADOS.

According to the National Census Register of Costa Rica, the CVCR harbors approximately 250,000 children between 0–4 years, another 250,000 between 5–9 years, and a total of one million individuals under the age of 20, with a slight preponderance of males in all age groups. (These numbers represent just under two-thirds of the totals for the entire country, as that is the estimated percentage of people living in the Central Valley). Assuming that published prevalence estimates in other countries apply to Costa Rica, as has been documented for Tourette's Syndrome and Bipolar Disorder [19], and taking a conservative value of 1 in 1,000 [2] as the prevalence estimate, there should be ~1,000 cases of autism between ages 0–20. Given that few cases will be seen before two or three years of age, and that we require Central Valley ancestry (not just birth) for entry into the study, the number of eligible cases may be anywhere from 300–500.

With the appropriate instruments and research-trained bilingual clinicians, we have begun recruiting autistic subjects and their parents from the CVCR for a genetic study. The first stage of the genetic study will be to perform highthroughput cytogenetic screening using a microarray-based Comparative Genomic Hybridization assay specially designed to detect microdeletions and duplications (microdels/dups), including sub-telomeric abnormalities, too small to be identified by standard karyotyping. Should we have a suitable sample size of cytogenetically normal cases after this evaluation, possibly augmented by samples from the genetically related population in Antioquia, we intend to perform a genome-wide single nucleotide polymorphism (SNP) screen to identify autism susceptibility genes. We also intend to use our samples to perform fine-mapping of autism loci identified by other investigators. Here we report complete clinical data gathered on a representative 35 cases recently assessed in the CVCR. Although this is a preliminary assessment, our findings related to the problems of IQ testing and recruitment in this population may be helpful to other Latin American investigators planning similar studies.

## Methods

This project was approved under the guidelines of the Ministry of Health of Costa Rica, the Ethical committee of the National Children's Hospital in San Jose, and the Institutional Review Board at Mount Sinai School of Medicine. These approvals remain active. All adult participants provided written informed consent. Parents of participating minors provided written informed consent; minors who were capable also provided signed assent.

### Translation of the ADOS

Dr. Valeria Nanclares completed a preliminary translation of the ADOS. She is the Autism Program Coordinator at the Pediatric Developmental Center, Advocate Illinois Masonic Medical Center, and also the translator of the ADI-R. Next, two professional translators read the document separately in English and Spanish and documented instances where the translation was weak or ambiguous. This approach avoids the introduction of synonyms and colloquialisms and is thus preferable to simple back-translation. Subsequently, native Spanish-speaking clinical experts from Latin America and Spain reviewed the document for content and accuracy of clinical terminology and achieved a final consensus with the original translator. The Latin American expert is Dr. Elina R. Manghi (ERM), who is also the lead best-estimator for this study. She is a clinical psychologist and the Clinical Service Director for the Family Clinic, Department of Disability and Human Development at the University of Illinois at Chicago. She has over 20 years of experience in the field of psychological testing and for the last 10 years has worked with developmental disorders, including autism. She is a certified off-site trainer for both the English and Spanish versions of the ADI-R and is certified for the research use of the ADOS in the United States. The expert from Spain was Dr. Amaia Hervas, who has worked extensively with Dr. Michael Rutter's group on various autism research projects.

### Ascertainment of cases

The Hospital Nacional de Ninos (HNN) is located in San Jose, the capital city of Costa Rica, and is the only major hospital for children in the CVCR. The HNN has an inpatient Neurodevelopmental Unit and recently added a small special education school providing services for children with mental retardation (MR) and developmental delay but not specifically autism. All four child psychiatrists serving the CVCR are also at the HNN. The leading clinician on our study, Dr. Patricia Jimenez Gonzalez (PJG), is Chief of the inpatient Neurodevelopmental Unit at the HNN and has an extensive private practice. She also has an active relationship with the Autism Parents' Association of Costa Rica, a group that is highly motivated to participate in research and promote community education. PJG has passed Dr. Catherine Lord's certification course for research use of the ADI-R and ADOS.

A recent study showed that the number of autism and autism spectrum disorder (ASD) cases being diagnosed in the United States increased precipitously following the introduction of Federal special education laws that included autism spectrum disorders as a disability category in 1991 [26]. The diagnostic criteria for autism were also broadened in DSM III-R in 1987 and DSM IV in 1994, and one study shows that both the changes in the formal diagnosis of autism, as well as the laws providing Federal support for children with ASD, can account for the increased incidence of this disorder in Olmsted County, Minnesota [27]. Therefore, it is not surprising that awareness of autism is only a recent phenomenon in Costa Rica, as this country lacks sufficient financial support for treatment of children with ASD. As a result, we began our recruitment with a list of 245 potential cases known to Dr. Jimenez from the HNN and her outpatient practice. These cases were selected because of documented developmental delay (cognitive/language and motor) and/or extremely disruptive behaviors characteristic of autism that lead parents to seek consultation in the first place. However, we have formulated a strategy to find the cases of high functioning autism, with normal or near normal IQ, that are not being seen by clinicians or treatment providers, or are being seen but not properly diagnosed. For instance, one study recently reported that an increase in the diagnosis of ASD between 1983 and 1996 appeared to be due to greater recognition of ASD in higher functioning children initially presenting with attention deficit hyperactivity disorder (ADHD) [28]. Another study surveyed the medical and psychiatric records of 101 Swedish children of normal intelligence (mean age 9 years, 8 months) diagnosed with PDD, in order to identify cases that might actually represent high functioning autism, and found that three-quarters had symptoms compatible with mild to severe ADHD or problems with attention and motor control [29]. These children also tended to be doing poorly in school or to be in special education programs despite their normal intelligence. Therefore, we will offer training to child psychiatrists, pediatricians, and special education teachers, under the auspices of the Costa Rican Education Ministry, to help them recognize potential cases of high functioning autism. ERM is actively involved in providing this training in Illinois. She will demonstrate this training model, which can be repeated several times a year. ERM has generated a large number of referrals for evaluation of autism at her bilingual Spanish Autism Clinic, which is part of the Family Clinic, Department of Disability and Human Development, University of Illinois at Chicago. We will also invite members of the Autism Parent Association of Costa Rica to participate in this training. We anticipate this training strategy will increase the awareness of high functioning autism, resulting in referrals for the project.

### Diagnosis of autism and autism spectrum disorders

Families of individuals with possible autism contact us, or are contacted by us after expressing interest in the study, and are formally asked to participate using established informed consent criteria. They are first screened by PJG using an abbreviated version of the ADI-R (Autism Screening Questionnaire) for the presence of symptoms consistent with a developmental disorder, as well as for pertinent medical information. All interviews and exams take place in the Neurodevelopmental Unit of the HNN. Possibly affected individuals and their parents are interviewed by PJG or Dr. Marietha Fallas Delgado (a pediatrician under her supervision) using the ADI-R. Either PJG or Sylvia Monge Monge, M.A., who has a master's degree in psychoeducation, administers the ADOS. Both of these assessments are videotaped for independent scoring by the best estimators.

An IQ score and an adaptive behavior score are required for the diagnosis of MR. Intelligence is assessed using age and developmentally appropriate instruments available in Spanish and currently in use in Costa Rica. However, a major problem with these IQ tests is that there are often no norms specific for individual Latin American countries. A few IQ tests have norms derived in Spain and Mexico, yet, according to Domingo Campos, Director, Instituto de Investigaciones Psicologicas de la Universidad de Costa Rica (Psychological Research Institute, University of Costa Rica), Costa Rica has no internal data of its own to assess potential scoring biases that might be introduced using foreign norms. The tests we use include the Bayley Scales of Infant Development II (1993) [30] (0–42 months developmental age), the McCarthy Scales of Children's Abilities (1972) [31] (2-1/2 to 8-1/2 years), the Leiter-R (1997) [32] (2 years to 20 years), the Wechsler Preschool and Primary Scale of Intelligence-Third Edition (2002) (WPPSI-III) [33], which is divided into two subtests for ages 2:6–3:11 years and 4:0–7:3 years, and the Wechsler Intelligence Scale for Children, Third Edition (1991) (WISC III) [34]. The IQ reflects how children answer verbal reasoning questions and respond to visual-spatial reasoning tasks. Because language and attention are impaired in children with autism, assessments of their IQs are notoriously unreliable. Therefore, we also administer the Vineland Adaptive Behavior Scales/VABS [35] to each proband. The VABS is a structured interview of the parent concerning observed communication and social and daily living skills at home and in the community. Both the IQ and VABS are administered by our psychologist, Licenciada Marcela Esquivel Pla., who was trained in these instruments by ERM.

#### Physical examination and laboratory testing

Each autistic proband undergoes a complete physical examination, including a neurological examination, an assessment for dysmorphic features, and a dermatological evaluation. Skin is carefully examined to look for signs of neurocutaneous disorders, including tuberous sclerosis (using a Wood's lamp) and hypomelanosis of Ito, as these disorders are strongly associated with autistic symptomatology. (Of note, one of the world's experts on hypomelanosis of Ito is doing a study at the HNN). Probands with gastrointestinal problems are sent to Internal Medicine for evaluation as at least one study has shown that an unusual complement-mediated lymphocytic colitis may be unique to severely affected autistic subjects [36]. All probands are karyotyped. Testing of auditory brainstem-evoked responses is requested in some cases, and computerized tomography scans are also available on some probands. Fragile X testing is done on all cases. Phenylketonuria (PKU) testing is also performed when required, as it was not instituted as a routine procedure in the CVCR until ~10 years ago.

#### Medical and social history

Please see the Clinical History form [see Additional file 1] for complete details of this evaluation. Sources of information for medical and social history include parents and medical records. In brief, we record parental occupational and educational history, followed by family neuropsychiatric history, obstetric data, including any adverse events during pregnancy and APGAR scores, the developmental and medical history of the proband, any medication and/or education the proband has received, and results of the physical exam and laboratory testing. As the family history of psychiatric disorders relies on parental report, it cannot be regarded as fully reliable. Therefore, we try to validate the diagnoses by asking about hospitalizations and medication treatments when possible.

In both the immediate and extended family we also specifically ask about the presence of language delay and learning deficits, which could be manifestations of the broader autism phenotype. We also ask about autoimmune disorders, as data suggests that at least some of the autistic phenotype may be induced by autoimmune mechanisms operating during pregnancy [37].

#### Verification of genealogy

Don Eduardo Fournier is a Costa Rican historian with extensive experience in tracing genealogies who collaborates on a majority of the genetic studies in Costa Rica. He screens the Civil Registry and, if necessary, birth, marriage, and death records in churches and civic centers to determine the birthplace of all grandparents and great-grandparents. Previous genetic studies in the CVCR have shown that probands whose great-grandparents were born in the CVCR will most probably be descendants of the original Spanish founder population. We require that cases have six of eight great grandparents born in the CVCR for inclusion in the study.

#### Best estimate diagnostic process

The ADI-R and ADOS are independently scored by our bilingual best-estimators, ERM or Pamela Bondy, M.S./CCC-L (PB). PB is a bilingual speech and language therapist with over 10 years of experience in the field of developmental disabilities. She was trained in the use of the ADI-R and ADOS by ERM. Discrepancies, if any, were resolved and final diagnoses reached after review of collateral historical information, clinical exam, and laboratory testing, according to standard best-estimate diagnostic procedure [38].

## Results

To date we have ascertained 76 subjects and enrolled 70 families in our study. One family later declined to participate because of the mother's health, two cases have been excluded by genealogy, one case was too retarded to determine a diagnosis, one case has RETT syndrome (AU043), and the other excluded case is an autistic child with Moebius syndrome (AU044). We are screening the RETT syndrome case for mutations in the *MECP2 *gene. The Moebius syndrome case offers fascinating insights into the etiological basis of autism and will be reported in detail in a subsequent publication. Extrapolating from the entire data set of 70 cases, the male to female ratio appears to be ~6:1. Our goal is to collect over 200 trios, with no major or minor cytogenetic abnormalities, suitable for genetic mapping.

As the diagnostic evaluation takes two to three appointments, we have only complete best-estimated data on 35 cases to date. Karyotypes ordered for cases AU025, AU031, and AU040, with family histories of MR, and for case AU001, who has a cleft palate, were all negative at ≥ 550 bands. Interestingly, case AU025 and case AU031 are related to each other by a distance of seven generations, and a connection with case AU040 has not been ruled out. Case AU008, suspected of Angelman's syndrome, had one normal karyotype, and testing for a chromosomal 15q11-q13 abnormality will be performed. The rest of the karyotypes are pending.

Table 1 in the text contains abbreviated descriptive statistics on the first complete 35 cases [see Additional file 2]. Ages range from 3 years to 13 years of age, and 31 of the 35 cases are male. Mothers and fathers of the autistic children have at least some high school on average. The average mother is a homemaker and the fathers are skilled laborers; overall, the socioeconomic status of the parents is low. The mean total score for the social domain of the ADI-R is 25.3 ± 5.4 in our sample; the nonverbal and verbal communication domain mean scores are 12.6 ± 1.8 and 17.8 ± 3.4, respectively; and the mean score for the repetitive behaviors and stereotyped patterns domain is 6.6 ± 2.3. The ADOS module scores reflect the severity of the ADI-R scores in all cases. Vineland adaptive behavioral profile composite scores range from 20–80, excluding the value of 127 for case AU029 with Asperger's, and the mean score is 48.5 ± 19.4. The majority of full-scale IQ scores are less than 50. Delayed onset of phrase speech is present in 24 of the 35 cases.

**Table 1:** is an abbreviated version of descriptive data obtained for each proband, including their sex and age at interview. Scores for each domain of the ADI-R are provided, as well as the full-scale IQ score and the Vineland Adaptive Behavior composite score. The superscript numerals next to the IQ score indicate the specific IQ test that was used.

Case	Social total	Communi-cation nonverbal total	Communi-cation verbal total	Behavior total	Age at first single words	Age at first phrases	Gender	Age at interview (ADI-R)	Full-scale IQ	Vineland Adaptive Behavior composite score
001	29		18	10	1	1	M	7.83	49^6^	37
002	29		20	5	0	0	M	5.67	<50^8^	53
003	28		25	6	0	1	M	6.75	87^6^	60
004	27	14		8	1	1	M	7.50	<50^5^	30
005	26		22	10	1	1	F	5.42	57^6^	51
006	9		20	5	1	1	M	6.25	67^7^	54
007	21		20	10	0	1	M	10.00	37^7^	20
008^1^	29	12		8	0	0	M	5.08	NA	45
009	27		19	9	0	1	M	8.08	34^7^	46
010	27		21	5	0	1	M	9.25	43^6^	35
011	27		16	6	0	1	M	10.50	NA	50
012	20	8	14	8	1	1	M	5.25	72^7^	70
013	29	14	16	6	0	0	M	4.33	<50^5^	44
014	29	13	19	6	1	1	M	6.50	<50^5^	51
015	30		14	9	0	1	F	5.03	<50^5^	46
016	28	12	20	12	1	1	M	13.00	60^6^	39
018	27	11		7	1	0	M	3.67	<50^5^	67
019	29		17	7	0	0	M	7.25	66^6^	63
025	28	12		8	0	0	M	7.83	<50^5^	24
027	24		18	6	1	1	M	4.25	<50^5^	55
028	29	14		3	0	1	M	5.42	<50^5^	47
029^2^	16		9	2	1	1	M	6.83	113^6^	127
030	27	13		5	1	0	M	5.66	<50^5^	43
031	29		15	4	1	1	M	7.08	70^6^	52
032	16	11		4	1	0	M	5.42	<50^5^	42
033	18	8		6	0	0	F	4.83	<50^5^	46
035	18		15	5	1	1	M	7.08	<50^5^	45
036	29	14		3	0	0	M	6.33	<50^5^	32
039	30	14		5	0	0	M	9.82	<50^5^	21
040	15		15	5	1	1	M	3.08	75^5^	80
049	29	14		9	1	1	M	13.25	<50^5^	20
050	21	13		6	1	1	M	2.92	pending	60
051	30	14		8	0	1	F	5.50	<50^5^	39
053	29		18	8	0	0	M	5.83	105^6^	55
056	28		21	7	?	1	M	6.00	56^6^	49
043^3^	22	12		5	0	0	F	4.83	NA	41
044^4^	27	11		2	1	1	F	5.75	NA	50

^1^Angelman's syndrome

^2^Asperger's syndrome

^3^Rett syndrome

^4^Moebius syndrome

^5^Bayley

^6^WPPSI

^7^Leiter

^8^McCarthy

NA indicates that the IQ test could not be completed because the child is too mentally retarded or was too distracted.

The Family History of Medical Disorders chart is available in the supplementary data [see Additional file 3]. AU033 developed epilepsy at the age of 2 years and 9 months, but is the only proband with this condition. Six of the cases have minor congenital defects, including a cleft palate, hip rotation, visual problems, megacisterna magna, and esophageal reflux/cardiomyopathy. Twelve of the cases have first- or second-degree relatives with language delay or learning deficits. Developmental delay or autism is present in the first-degree or second-degree relatives of four cases. Four of the cases have a second-degree relative with non-specific MR, one case has a second-degree relative with Down's syndrome, five cases have a second-degree relative with epilepsy, and one case has a sister with a minor congenital defect (cleft palate). Six cases have first-degree relatives with a major psychiatric disorder, including schizophrenia and major depression. No cases have tested positively for Fragile X or phenylketonuria, and no history of autoimmune disorder was documented for any case.

We have obtained extensive obstetric data on our cases and will be reporting it in full in a subsequent publication. In brief, although only 4 of the mothers were older than 35, 21 of them had obstetric complications and/or the infant had an APGAR score less than 6 at one minute. Fully twenty of the cases were the product of difficult labor, resulting in fetal distress and a caesarean section in eight instances.

## Discussion

On the whole, our first cases have mean ADI-R scores comparable in severity to those of other autism genetic studies, including a sample from the Autism Genetic Resource Exchange (AGRE) (N = 364 cases from 212 sibships) [12] and a sample of 43 sibships from the International Molecular Genetic Study of Autism Consortium [16]. IQ and adaptive behavioral scores were not reported for the AGRE cases, but our mean VABS composite score is 48.5 ± 19.4 versus a mean of 47.44 ± 16.15 for the IMGSAC cases. However, it is clear that the cases of autism known to the HNN are more severe than those seen in other non-Hispanic populations studied recently, although perhaps not before 1991.

Eighty-three percent of our cases are mentally retarded, while 60% have IQs <50. In contrast, epidemiological data from a range of countries, although none of them Latin American, estimate that 75% of autistic individuals have IQs <70 and 40% have IQs <50 [2]. However, a closer look at all the major epidemiological studies to date, reviewed by Fombonne in 2003, shows that the median prevalence of autism was only 4.4/10,000, and the reported percentages of normal IQs were, in general, lower in studies done before 1991 when there was also less awareness of autism. In contrast, the median prevalence of autism is 12.7/10,000, and the percentages of normal IQs is increasingly higher, in studies done more recently. This is in part due to a broader definition and recognition of autism, but also, at least in the United States, it is possible that early intervention services, instituted over 15 years ago, have improved the cognitive performance of autistic children. For instance, some studies report that cases of autism with low IQs can achieve marked improvements in intelligence, language, learning, or visuo-spatial skills [39,40] with a minimum of two years of intervention [41], although others feel that outcome measures have not been properly evaluated [42]. Costa Rica now provides some special education services; however, these are not specific to children with autism and are not offered to children more than once weekly. Moreover, teachers are not trained in autism interventions, and many children complete only primary education.

We must also consider the possibility that the IQs of our cases are underestimated due to the use of psychometric tests without Costa Rican norms. A recent study done in Argentina illustrates the effects of low socioeconomic status and lack of internal norms on IQ testing results. This group set out to perform a longitudinal study of the effects of poverty and malnutrition on the psychological development of children in a province of Buenos Aires [43]. The study took place from 1984 to 1995 and followed 100 children of low socioeconomic status with or without malnutrition. Cases between ages 0–2 with malnutrition but without other neurological or medical deficits were ascertained from an ambulatory Nutritional Rehabilitation Unit of a local hospital and matched with well-nourished cases of the same age and sex from the same socioeconomic background. A pediatrician, a pediatric neurologist, a psychologist, and an anthropologist performed the evaluations. Ninety-two children were reassessed at 2 years, and 44 were reassessed at the 10-year point. Malnutrition was rectified in that group during the two-year interval between assessments. For the initial evaluation, the group used the Escala de O. Brunet-I. Lezine, which is used to assess global psychomotor development of 0–2 year olds. At the next two time points, they used the Terman-Merril (Forma L-M) intelligence test to obtain IQs. This test was normed on children in the United States, and there were no norms available for Argentina.

At the first evaluation, the malnourished children did have significantly lower developmental quotients than the well nourished group, the majority of which had normal ratings. However, at time point 2, after which the malnourished children had been treated, there was no difference between groups, but both were lower than the standard. At time point 3, when the children were ~10 years of age, there was again no difference in IQ between groups, but ~50% of both groups had IQs = 70. The authors of the study drew two major conclusions from these results. The first was that poverty and lack of early intellectual stimulation had profound effects on IQ, even without malnutrition, and that these effects became more prominent with time. The second conclusion concerned the use of IQ tests without norms reflecting the general population of Argentina. The raters felt that the IQ scores of both groups were probably somewhat underestimated because the norms they were comparing them to were derived from children with much higher socioeconomic status. The raters were very experienced in administration of the IQ tests, which were commonly used at universities and elsewhere in Argentina, so misuse of the tests was not an issue. Unfortunately, Latin American governments do not invest in psychological research, and the corporations that sell tests in the United States don't see Latin America as a strong market and thus put no money into norming translated tests. (We wish to note that Argentina now has its own norms for some IQ tests but not all.)

As further evidence of problems with IQ testing, and perhaps ascertainment bias, in the CVCR, we note that in genetic studies of Tourette's Syndrome and ADHD ongoing in the CVCR, 20–25% of cases evaluated for either phenotype have been excluded because of MR (PJG, unpublished data). In contrast, 3.9 % of children with Tourette's Syndrome (TS) had MR (range 1–14%) in a large-scale analysis of this disorder in 22 countries (no specific data for Spanish-speaking countries) [44]. Additionally, although children with ADHD have, on average, IQs 7–12 points lower than comparison children, the rate of MR observed in the CVCR is certainly not characteristic of ADHD in North America [45,46]. Of course, an ascertainment bias towards severe cases could also explain the CVCR IQ data for ADHD and TS. However, data concerning the prevalence of epilepsy in autism and ASD also suggests that the IQs of our subjects may be underestimated. For example, severe MR is often seen in cases of autism with a comorbid medical disorder. Early seizures, before two years of age, were present in 60% of this population in one study, and 20% of autistic subjects with severe MR but no comorbid medical disorders experienced seizures before the age of five [47]. As the mean age of our probands is 6.8 years and only one of them has experienced seizures, it seems less likely that autism is secondary to other medical factors in the majority of our cases. Finally, the median male to female sex ratio of 11 previous epidemiological studies, wherein the majority of cases were moderately to severely retarded, was 1.9:1, versus 5.75:1 for studies of predominantly normal IQ probands [2]. We also observe a family history of MR in 4 of our cases, but our male to female sex ratio is ~6:1 (also true for the 70 cases collected to date).

Accurate assessment of IQ is important, as data suggests that that there may be different genetic and environmental determinants for autism based on level of cognition, although cases of autism with low IQ may still have a strong genetic basis. For instance, Baird & August [48] observed a familial loading in cognitive impairment confined to autistic probands with severe or profound MR. To determine if autism with severe MR was a distinctly different autistic endophenotype associated with increased familial cognitive impairment, another group [49] studied 47 autistic probands with an IQ <50 and contrasted their findings with a sample of 99 autistic subjects, the majority of whom had IQs >50 [6]. Importantly, they found that increased loading for ASD was comparable in both samples, supporting prior evidence that the same genetic liability applies to autism and ASD within families, regardless of IQ level. Interestingly, a recent study also shows that co-occurrence of lower IQ and ADHD reflect common genetic determinants and not necessarily the presence of a primary medical or neurological disorder [46].

We do note frequent obstetric complications in our sample and are researching the rates of these complications in the CVCR hospitals for comparison. We are also looking at factors such as geographic distance from a hospital, which might contribute to suboptimal labor and delivery. However, recent data suggests that these complications are commonly associated with autism and ASD. A large-scale epidemiological study of autism in Western Australia found that cases diagnosed with autism, as well as their unaffected siblings, had more obstetric complications than a control group [50]. The authors suggest that the increased prevalence of obstetric complications among autism cases is most likely due to underlying genetic factors, possibly exacerbated by environmental stressors.

There are many possible underlying cytogenetic or other medical disorders that may be accompanied by autistic behavior, and the presence of severe MR can make those cases suspect even if karyotypes are normal. Disturbingly, a group of investigators recently reported that they found microdups/dels, as well as other genetic abnormalities, in 272 cases from the Paris Autism Research International Sibpair (PARIS) sample that had already been included in genetic linkage and association studies [51]. These include a 22q11 deletion without the associated features of DiGeorge/Velocardiofacial syndrome in two autistic brothers with severe MR, and their mother, as well as duplications of 15q11-13 (in two different singletons) and of 17p11.2 and 7q11.2. Therefore, we have developed a collaboration to screen our cases using microarray-based Comparative Genomic Hybridization technology. We will hybridize our samples to a whole genome array containing 2632 BAC (bacterial artificial chromosome) clones at a resolution of ~1 Mb spanning the 22 autosomes and the X and Y chromosomes. In this way we will find any known and novel microdups/dels, as well as subtelomeric abnormalities, in our cases before we proceed with any genetic association analyses. Thus, we feel confident that our cases, regardless of IQ, will be as free of genetic abnormalities as can be determined given current technology.

## Conclusion

We have successfully used the Spanish versions of the ADI-R and ADOS to diagnose autism for genetic study in the CVCR. The majority of the cases reported here have IQ <50. This reflects an ascertainment bias, yet it is also possible that these scores are artificially low. Nevertheless, we are most likely missing cases of autism with normal or near normal IQ due to a lack of government-sponsored services. Therefore, we are implementing a strategy, used successfully by one of our clinical experts to find both English-speaking and Spanish-speaking higher functioning autistic subjects in the United States, to ascertain these cases. We believe that similar problems with IQ testing and the need for specific strategies to find high functioning autism cases will be important issues to address for studies of autism planned or ongoing in other Latin American countries.

## Competing interests

The author(s) declare that they have no competing interests.

## Authors' contributions

LAM secured the funding for the study, played a major role in designing the study, contributed to the data analysis, and wrote the manuscript.

PJG is the leading clinician in Costa Rica and performed the majority of the diagnostic interviews using the ADI-R and ADOS, as well as the neurological and medical evaluations. She contributed to the study design and to the data analysis and ensures compliance with the Bioethics Committee at the HNN.

ME coordinated the study in Costa Rica and contributed to the data analysis. She administered informed consents and IQ tests, arranged appointments with subjects.

ERM is the lead best estimator for our study and performed the majority of data evaluations. She trained the Costa Rican team in the use of current psychological instruments. She also contributed to the manuscript.

MFD performed diagnostic interviews using the ADI-R and contributed to the data analysis.

SSM assisted PJG in performing the ADOS and contributed to the data analysis.

EF traced the genealogies of the probands and is responsible for establishing familial relationships between the cases as well as providing population data for the CVCR essential to the manuscript.

PB performed best-estimate data evaluations and contributed to the data analysis.

KC coordinated the study in the USA, entered diagnostic information into a database for future statistical analysis, contributed to the manuscript and ensured compliance with the Institutional Review Board at Mount Sinai School of Medicine.

All authors read and approved the final manuscript.

## Pre-publication history

The pre-publication history for this paper can be accessed here:



## Supplementary Material

Additional File 1 Formulario de historia clinica This is the clinical history form that we designed in Spanish. The table begins with parental occupational and educational history followed by family neuropsychiatric history, obstetric data, including APGAR scores, the developmental and medical history of the proband, education the proband has received and results of the physical exam and laboratory testing. Click here for file

Additional File 2 Results of Diagnostic Evaluations for Autism This table contains scores for the individual scores subdomains of the ADI-R, the ADOS scores, the IQ scores and information regarding the parents' occupations and education. Click here for file

Additional File 3 Family History of Medical Disorders This table contains details of the family and personal medical history for each proband. We specifically asked about the presence of autism, language delay, learning deficits, MR, epilepsy or other neurological abnormality, cerebral palsy, major psychiatric disorders and deafness. For instance, if a first cousin on the maternal side had epilepsy, we typed 'M first cousin' in the appropriate box. The letters 'M' or 'P' indicate whether the relative is on the maternal or paternal side, respectively. If the mother had a complication during pregnancy such as bleeding or a urinary tract infection, this is noted with an x in the appropriate box. APGAR scores are recorded, as well as the presence of any congenital defects in the proband. Click here for file
